# Giant Piezoelectric Output and Stability Enhancement in Piezopolymer Composites with Liquid Metal Nanofillers

**DOI:** 10.1002/advs.202304096

**Published:** 2023-09-13

**Authors:** Jingyan Liu, Shi Zeng, Mingrui Zhang, Juan Xiong, Haoshuang Gu, Zhao Wang, Yongming Hu, Xianghui Zhang, Yi Du, Long Ren

**Affiliations:** ^1^ Hubei Key Laboratory of Ferro & Piezoelectric Materials and Devices School of Microelectronics Hubei University Wuhan 430062 P. R. China; ^2^ Center of Quantum and Matter Science and School of Physics Beihang University Beijing 100191 P. R. China; ^3^ State Key Laboratory of Advanced Technology for Materials Synthesis and Processing International School of Materials Science and Engineering Wuhan University of Technology Wuhan 430070 P. R. China

**Keywords:** liquid metals, liquid‐solid interface, piezoelectric composites, piezoelectric energy harvester

## Abstract

Integrating nanomaterials into the polymer matrix is an effective strategy to optimize the performance of polymer‐based piezoelectric devices. Nevertheless, the trade‐off between the output enhancement and stability maintenance of piezoelectric composites usually leads to an unsatisfied overall performance for the high‐strength operation of devices. Here, by setting liquid metal (LM) nanodroplets as the nanofillers in a poly(vinylidene difluoride) (PVDF) matrix, the as‐formed liquid‐solid/conductive‐dielectric interfaces significantly promote the piezoelectric output and the reliability of this piezoelectric composite. A giant performance improvement featured is obtained with, nearly 1000% boosting on the output voltage (as high as 212 V), 270% increment on the piezoelectric coefficient (*d_33_* ∼51.1 pC N^−1^) and long‐term reliability on both structure and output (over 36 000 cycles). The design of a novel heterogenous interface with both mechanical matching and electric coupling can be the new orientation for developing high performance piezoelectric composite‐based devices.

## Graphical abstract

Liquid metal nanodroplets are introduced into P(VDF‐TrFE) matrix to construct a liquid‐solid piezoelectric nanocomposite. The optimized liquid metal‐PVDF nanocomposites with polar *β*‐phase content of 81.5% demonstrate an impressive piezoelectric coefficient (*d_33_* ∼51.1 pC/N) and maximum output voltage of ≈212 V. The flexible device presented long‐term stability and realized the self‐powered monitoring and smart analysis of human limbs and facial movements.

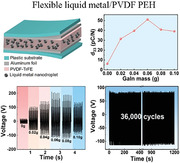

## 1 Introduction

Piezoelectric materials, which can directly convert mechanical energy to electrical energy, offer broad application prospects for energy harvesters, sensors and actuators.^[^ ^1^ ^]^ Ceramic‐based piezoelectric materials have been well‐developed, but they are lacking structural flexibility that hinders their applications in lightweight and flexible devices.^[^ ^2^ ^]^ Piezoelectric polymers (piezopolymer, e.g., poly(vinylidene fluoride) (PVDF) and its copolymers) possess good flexibility and easy processibility, demonstrating advantages toward wearable piezoelectric devices. However, their low piezoelectric output and weak ferroelectric stability largely limited their practical performance. One effective solution to this limitation is increasing the proportion of the electroactive polar phase (i.e., *β*‐phase of PVDF) by incorporating nanofillers into the piezopolymer matrix to regulate the transformation of the polymer conformation.^[^ ^3^ ^]^ Nevertheless, the mechanical mismatch between the frequently‐used rigid solid nanofillers and soft polymer matrix usually results in an interfacial spatial discontinuity during repeatable strain implementation, which would deteriorate the output performance and hinder the durability of the device. Therefore, exploring a new type of polymeric piezoelectric nanocomposites is essential for the development of flexible piezoelectric devices with high performance and well stability.

Compared to rigid solid nanofillers, integrating soft objects with good deformability into a piezopolymer matrix is conducive to forming continuous interfaces and enhancing interfacial stability.^[^ ^4c^ ^]^ Among all soft materials, liquid materials in the form of droplets have almost infinite deformation capability. Incorporation of liquid phase into a polymer matrix can create a well‐matched liquid‐solid interface, and such interfaces are reliable due to the adaptive shape‐shifting ability of the filled droplets during high‐frequency vibrations.^[^ ^5^ ^]^ However, it is challenging to develop a suitable type of liquid droplets as stable and effective nanofillers for the fabrication of high‐performance piezopolymer composites. For example, aqueous or organic droplets would tend to be eliminated or transformed during the solvent evaporation process required for the crystallization of PVDF‐based piezopolymer.

Herein, we demonstrate a novel liquid‐solid interface featuring piezoelectric nanocomposites by directly using Ga‐based liquid metal (LM) nanodroplets as the nanofillers in the PVDF‐based matrix. Ga‐based LMs are new emerging conductive liquid materials, possessing high electrical/thermal conductivity, excellent fluidity, and good biocompatibility. On account of their very low saturated vapor pressure and immiscibility with most organic/aqueous solvents, the Ga‐based LMs have recently developed as a good conductive filler for developing metal/polymer‐based flexible electric circuits.^[^ ^6^ ^]^ In this work, the GaIn (eutectic alloy, a typical LM alloy) nanodroplets (NDs) obtained via the ultrasonication process were embedded into the piezoelectric Poly(vinylidene fluoride‐trifluoroethylene) (PVDF‐TrFE) matrix, forming GaIn NDs/PVDF‐TrFE composites. Although the GaIn nanodroplet is conductive, a thin native oxide layer would form at the surfaces, which can be considered as an electrostatic interaction center with a dielectric layer. It was revealed that such a configuration of nanofillers can facilitate the formation of the polar *β* phase in PVDF‐TrFE, and the thickness of this dielectric oxide layer could affect the final contents of the *β* phase and then regulate the piezoelectric output. More importantly, the structure of GaIn NDs/PVDF‐TrFE composites including the internal liquid‐solid interfaces is highly stable due to the LM nanofillers can adaptively shape‐shift with the deformation of the polymeric matrix. Specifically, the piezoelectric film composed by optimized GaIn NDs/PVDF‐TrFE composites exhibits an enormously high piezoelectric coefficient (*d_33_*) of 51.1 pC N^−1^, which is the highest among the reported values for the conductive‐filler‐enhanced PVDF‐TrFE based piezopolymer composites. The piezoelectric output can reach up to 212 V, nearly 10 times in comparison with the pristine PVDF‐TrFE based composites, which can monitor human limb and facial muscle movements during speaking and even simultaneously lighting 60 commercial LED lights. In particular, the output performance has negligible reduction after 36 000 cycles, manifesting excellent operation stability and durable performance of this LM‐piezopolymer composites system.

## 2 Results and Discussion

**Figure 1:**
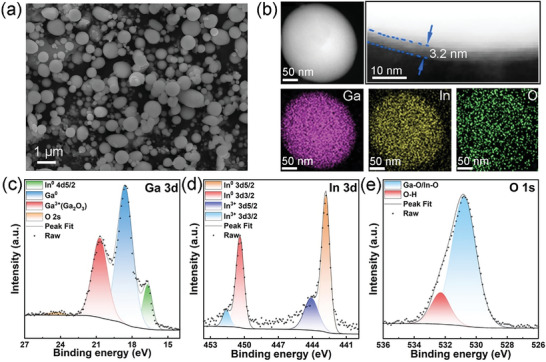
a) SEM image, b) HAADF‐STEM image and EDS mapping images. High‐resolution XPS spectra of c) Ga 3*d*, d) In 3*d* and e) O 1*s* of GaIn NDs.

The SEM image of the GaIn NDs prepared by ultrasonicating GaIn LMs in DMF is shown in **Figure** **1**a, which the GaIn NDs exhibit round spheres and the average size is in the range of 200–800 nm. The TEM characterizations further identify the component of the as‐prepared GaIn NDs. The HAADF‐STEM image and EDS mapping images of the GaIn ND (Figure 1b) also suggest that Ga, In and O elements are evenly distributed. In addition, a thin oxide layer with a thickness of ≈3.2 nm as the shell layer of GaIn ND can be observed in the partially enlarged view of a typical particle edge. It is noteworthy that, as illustrated in characterization results (Figure S2a and S2b, Supporting Information) of the GaIn nanoproducts prepared by sonication in water (this sample is denoted as W‐GaIn NDs), the compositional distribution of the oxides layer is more obvious and the thickness of such shell layer is over 10 nm. It demonstrates the oxides layer of the GaIn NDs can be regulated by changing the sonicating surroundings.

For the purpose of identifying the chemical binding as well as the oxidation states of the constituent elements of GaIn NDs, X‐ray photoelectron spectroscopy (XPS) was employed. As shown in the fully scanned spectrum in Figure S3 (Supporting Information), the appealing peak signals can be attributed to Ga, In, and O elements. The deconvolution of the high‐resolution spectrum for Ga in Figure 1c reveals the dominated existence of metallic Ga (≈18.8 eV) along with the co‐existence of Ga^3+^ oxide state (≈20.7 eV). The peak at 16.9 eV could be attributed to In 4*d*.^[^ ^7^ ^]^ The peaks at 450.4 and 442.8 eV in In 3*d* spectrum are respectively ascribed to In 3*d*_3/2_ and In 3*d*_5/2_, indicating the metallic state of indium, as shown in Figure 1d.^[^ ^8^ ^]^ The peak at 530.8 eV in the high‐resolution spectrum of O 1*s* in Figure 1e was an indication of the oxygen anions in Ga_2_O_3_ configuration.^[^ ^9^ ^]^ It's noted that no peak associated with adsorbed oxygen was observed in the O 1*s* spectrum, which further verifies the obtained GaIn NDs are actually GaIn@Ga_2_O_3_ core‐shell structures. It should be noted that, the ratio of Ga^3+^/(Ga^3+^+Ga^0^) (according to the related XPS spectra in Figure S2c–f, Supporting Information) in the W‐GaIn NDs sample is obviously higher than that in GaIn NDs sample from ultra‐sonication in DMF, which further confirms only a very thin oxide layer can form at the surface of GaIn NDs during the sonication in DMF surroundings.

**Figure 2:**
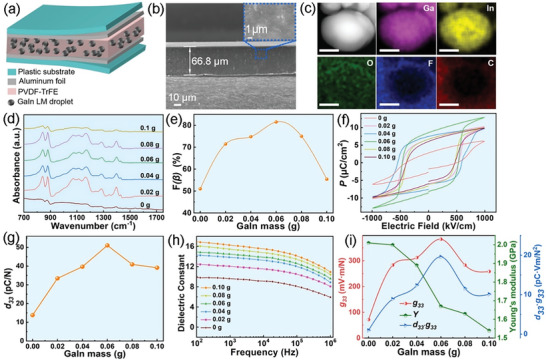
a) Schematic illustration on the configuration of the GaIn NDs/PVDF‐TrFE composites based PEH. b) The cross‐sectional SEM image of the GaIn NDs/PVDF‐TrFE membrane (the content of GaIn NDs is 0.06 g). c) HAADF‐STEM image and EDS mapping results of a microsize cross‐sectional area in the composite membrane (the content of GaIn NDs is 0.06 g). Scale bars: 100 nm. d) FT‐IR spectra, e) the variation plot of calculated *β* phase contents, f) P‐E loop, g) d_33_, h) dielectric constant, and i) g_33_, Young's modulus (Y) and *g_33_* ·*d_33_* of the GaIn NDs/PVDF‐TrFE composite membrane embedded with different mass contents of GaIn NDs.

By dispersing the obtained GaIn NDs into the PVDF‐TrFE precursor solution, a flexible GaIn NDs/PVDF‐TrFE composite membrane can be formed after the solvent evaporation and removal process. During this process, the VDF monomer would be polymerized and varying phases (e.g., polar *β* phase) of PVDF may be formed. **Figure** **2**a schematically demonstrated the construction of the corresponding piezoelectric energy harvester (PEH) device, in which the flexible LM/PVDF‐TrFE composite membrane was sandwiched between two Al foil electrodes. The cross‐sectional SEM image of the GaIn NDs/PVDF‐TrFE composite (Figure 2b) suggests the uniform distribution of GaIn NDs as the liquid nanofillers and the thickness of the composite membrane is ≈66.8 µm. The XRD analysis of the composites (Figure S4, Supporting Information) illustrates the formation of the polar *β* phase of PVDF‐TrFE, which is evidenced by the observation of the prominent diffraction peak appeared at 19.9° well‐indexed to (110) and (200) planes reflection of *β* phase PVDF.^[^ ^10^ ^]^ The diffraction peaks ≈31.7° and 35.2° for Ga_2_O_3_ are also found.^[^ ^11^ ^]^

In order to clarify the microstructure of the unique liquid‐solid heterogeneous configuration, scanning transmission electron microscopy (STEM) characterization equipped with energy‐dispersive X‐ray spectroscopy (EDS) analysis was conducted on a nano‐slice from a randomly selected area of the GaIn NDs/PVDF‐TrFE composite membrane. As demonstrated in the high‐angle annular dark‐field (HAADF) STEM image and EDS mapping results of this nano‐slice (Figure 2c), an individual GaIn ND (diameter ≈200 nm) composited with evenly distributed Ga and In elements is embedded in the polymer matrix. A small signal of O element is also found on the surface of such GaIn ND. And signals of both F and C elements indexed to the chemical constitution of PVDF‐TrFE matrix are observed in the corresponding background. The interesting thing is, the distribution of F elements is more intensive at the interface between the GaIn ND and matrix, which is usually observed due to the directional distribution of organic group containing elements F on the surface of nanofillers.^[^ ^12^ ^]^ The above results offered direct evidence supporting that the introduction of the LM nanofillers could endow the alignment of CF_2_/CHF dipoles of PVDF‐TrFE on the surface of the GaIn ND with a thin oxides shell, and then facilitate the further all‐trans conformation of *β* phase. A similar phenomenon of F element enrichment, but with a lower enrichment degree, can also be found in the PVDF‐TrFE composite membrane embedded with the W‐GaIn NDs (Figure S5, Supporting Information), indicating the alignment of CF_2_/CHF dipoles may be weaker on the surface of the GaIn NDs with a thick oxides shell.

The proportion of the nanofillers in the matrix is also a key parameter that affects the formation of the active *β* phase or the output performance of the piezoelectric nanocomposites. The surface morphologies of the GaIn NDs/PVDF‐TrFE nanocomposites with different weights of GaIn were characterized by FESEM and shown in Figure S6 (Supporting Information). The well‐dispersed isolated GaIn NDs in the PVDF‐TrFE matrix can be clearly observed when the weight of GaIn is in the range of 0.02–0.06 g as shown in Figure S6b–d (Supporting Information). When the GaIn mass is more than 0.06 g, some bigger bright regions were observed in Figure S6e,f (Supporting Information) indicating the aggregations of GaIn NDs in the PVDF‐TrFE matrix. As too many GaIn droplets were composited in PVDF‐TrFE, it is easy to merge due to the shorter distance between each other. The FT‐IR measurement was performed to identify the polymorph forms of PVDF‐TrFE and explore the effect of GaIn NDs loading mass. As displayed in Figure 2d, the absorbance peaks at 765 and 795 cm^−1^ were attributed to the nonpolar *α* phase, while the vibrational bands occurring at 840 and 1279 cm^−1^ corroborate the *β* phase in PVDF‐TrFE.^[^ ^10a^ ^]^ The effect of GaIn droplet loading mass on the percentage of the *β* crystals was calculated and plotted shown in Figure 2e by the Lambert‐Beer law:

(1) $$F\;\left(\beta \right)=\frac{A_{\beta }}{\left(\frac{K_{\beta }}{K_{\alpha }}\right)A_{\alpha }+A_{\beta }}\;$$

Here, *A_α_* and *A_β_* are the absorbance intensities at 765 and 840 cm^−1^, respectively. The values of *K_α_* and *K_β_* are 6.1 × 10^4^ and 7.7 × 10^4^ cm^2^ mol^−1^, respectively, representing the absorption coefficients at the corresponding wavenumbers.^[^ ^4a^ ^]^ It can be found that the percentage of *β* phase in GaIn NDs/PVDF‐TrFE composites increased significantly with the increasing mass of GaIn filler and obtained a maximum value of 81.5% for 0.06 g GaIn droplet filling that is 30% higher than the pure PVDF‐TrFE. And then the *β* phase fraction reduces with the GaIn NDs proportion further increasing. The agglomeration of GaIn NDs with a filling mass over 0.08 g becomes clear in the matrix (as illustrated in Figure S6e,f, Supporting Information), which implies the aggregation of LM nanofillers would lead to the decline of *β*‐phase. The above results and analysis clarify that effective filling and evenly distribution of LM nanodroplets with thin oxides shell is beneficial to enhance the percentage of polar *β* phase in the piezoelectric PVDF‐TrFE matrix.

As a significant characterization tool for evaluating the polarization state, the *P‐E* hysteresis loops of GaIn NDs/PVDF‐TrFE composite films with different GaIn masses are illustrated in Figure 2f. All the *P*‐*E* loops exhibit a large coercive field and high remnant polarization *P_r_*, demonstrating the ferroelectric property of all the samples and there is a marked uptick of the remnant polarization *P_r_* after filling GaIn NDs. Moreover, the *P_r_* value increased from 2.78 to 8.53 *u*C cm^−2^ by increasing GaIn droplet nanofillers to 0.06 g. The enhanced remnant polarization can be attributed to the strengthened local electric field by introducing the conductive liquid GaIn in the poling process.^[^ ^13^ ^]^ However, more addition of GaIn NDs results in decreased *P_r_* that is on account of the more conductive path, increased electrical leakage, and corresponding ferroelectric loss in the GaIn NDs/PVDF‐TrFE composite.^[^ ^3^, ^14^ ^]^ Furthermore, Young's modulus (*Y*) of the GaIn NDs/PVDF‐TrFE with various masses of GaIn particles are measured as 2.01, 2.00, 1.89, 1.67, 1.63, and 1.54 GPa, respectively. Different from introducing the PZT or BaTiO_3_ ceramic fillers, the addition of liquid GaIn led to the declined *Y* value due to its fluidity.^[^ ^15^ ^]^ And the piezoelectric coefficient was calculated by the formula *d_33_* = ‐*P_r_*/*Y*. The value of *d_33_* goes rising sharply with the addition of GaIn NDs and is maximized at 51.1 pC N^−1^ for 0.06 g GaIn fillers in the composite film, which is 2.7 times higher in comparison to that of pristine PVDF‐TrFE film (Figure 2g). It is especially concerning that the *d_33_* value of ≈51.1 pC N^−1^ of 0.06 g GaIn NDs/PVDF‐TrFE based PEH is the best one so far in conductive filler based PEHs within our knowledge.

Nevertheless, the energy conversion efficiency of the piezoelectric composite film is not entirely dependent on *d_33_*. To get insight into the effect of the liquid GaIn fillers on the piezoelectric output performance of the PEH, the dielectric constant (*ε_r_*) and piezoelectric voltage coefficients (*g_33_*) of GaIn NDs/PVDF‐TrFE composites were also investigated. It can be found from Figure 2h that the value of *ε_r_* of GaIn NDs/PVDF‐TrFE composites decreases as the increasing frequency of the alternating electric field and filling mass of GaIn particles. The tendency is similar to the reported literature based on the percolation theory that is owing to the strong polarization effect of conductive GaIn droplets on PVDF‐TrFE. Further, the *g_33_* is calculated by *g_33_* = *d_33_*/(*ε_r▪_ε_0_*), where *ε_0_* is the vacuum permittivity.^[^ ^16^ ^]^ As illustrated in Figure 2i. the value is greatly enhanced with the peak of 383.5 mV m N^−1^ at 0.06 g and then fell back by increasing the GaIn filler mass. Importantly, the mechanical‐electrical transduction coefficient (*d_33_* ·*g_33_*) is the critical parameter for PEH device, which was also calculated and plotted in Figure 2i. Although Young's modulus descends with more E‐GaIn, the value of *d_33_* ·*g_33_* presents a significant increasing tendency in the initial after introducing GaIn NDs fillers that was largely due to the sharp enhancement of *d_33_*. Its trend is in line with the tendency of *d_33_* and *g_33_*, and reaches maximums with optimal GaIn filling mass is ≈9.3 times that of the pristine PVDF‐TrFE film (19.5 for 0.06 g GaIn NDs/PVDF‐TrFE and 2.1 for PVDF‐TrFE). Notably, in comparison with the control W‐GaIn NDs/PVDF‐TrFE, the calculated *β* phase content and *d_33_* values based on FTIR spectra and *P‐E* loops (Figure S7a–d, Supporting Information) are lower than the case for GaIn NDs/PVDF‐TrFE with GaIn nanofillers prepared by ultrasound in DMF solution. Such a decrease should take into consideration the difference of the Ga_2_O_3_ shell layer thickness formed by ultrasonic in DMF solution and deionized water that will be explained and discussed in detail in the following section.

**Figure 3:**
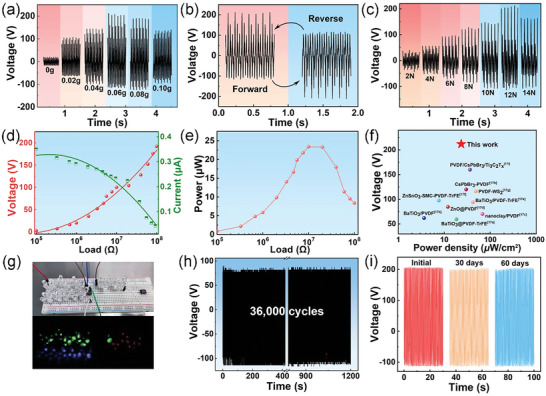
a) The output voltage of the GaIn NDs/PVDF‐TrFE based PEH with different mass of GaIn particles under 12 N tapping force. The output voltage b) in forward or reverse connection mode, c) with various tapping force. d) The output voltage and current evolution and e) corresponding output powers versus external load resistors of 0.06 g GaIn NDs/PVDF‐TrFE based PEH. f) Comparison of the output voltage and power density of different PVDF composited films based PEHs. g) Commercial LED bulbs illuminated by the GaIn NDs/PVDF‐TrFE PEH. Durability test of the PEH device: h) 36 000 cycles (at 30 Hz for 20 min with 10 N) and i) 60 days later (with 12 N at 30 Hz).

**Figure** **3**a shows the output voltage of GaIn NDs/PVDF‐TrFE PEHs with different masses of GaIn nanofillers under compression of 12 N with a frequency of 30 Hz. It is obvious that the mass of GaIn nanofillers creates an essential effect on the output performance of the PEHs. The voltage increases first and then dropped, which obtained a maximum of 212 V at 0.06 g. The value is ≈9.77 times that of the PEH based on pure PVDF‐TrFE, indicating the superb performance of PEHs with GaIn NDs filler. Particularly, the output voltage of W‐GaIn NDs/PVDF‐TrFE based PEHs with different masses of GaIn NDs presented significantly lower signal intensity (Figure S8, Supporting Information) owing to the lower *d_33_* value. As illustrated in Figure 3b, the GaIn droplet filler based PEH delivers alternating output signals when the device switches to the reverse connection, confirming the validity of piezoelectric energy conversion behavior. It is highly necessary to evaluate the performance of the PEHs with external impact force in practical application requirements. The output piezoelectric signals are plotted in Figure 3c by applying varying impacting forces ranging from 2 to 14 N. The piezoelectric output voltage augment was clearly observed from 44 to 212 V by increasing the force from 2 to 12 N, which is resulting from the strong piezoelectric polarization by the greater impacting force. A slightly reduced output for 14 N could be attributed to the recovery deformation failure at the frequency of 30 Hz. Next, we estimated the electrical outputs of our best PEH by changing the external load resistance from 100 KΩ to 100 MΩ by 12 N with a tapping frequency of 30 Hz. The instantaneous output power was calculated by the relation, *P* = *U_R_* ·*I_R_*, where the *U_R_* and *I_R_* were acquired from the external load resistances. As shown in Figure 3d, the output voltage increased from 2.4 to 192.3 V, while the output current shows the opposite tendency decreasing from 0.35 to 0.04 µA due to the ohmic loss. The maximum instantaneous output power was achieved at the load resistance of 23.4 µW for 0.06 g GaIn droplet filled PEH (Figure 3e).

Actually, the prominent output voltage over 200 V is hardly available in PVDF composited films based on PEHs, as shown in the projected chart in Figure 3f.^[^ ^17^ ^]^ For instance, S. Lim et al achieved open circuit voltage (≈62 V) in BaTiO_3_ nanorods‐contained PVDF nanofibers based PEH.^[^ ^17b^ ^]^ The PVDF‐based PEH composited with CsPbBr_3_ exhibits the peak output open circuit voltage of 120 V.^[^ ^17h^ ^]^ Additionally, the best output power of ≈23.4 µW in our work reveals the satisfactory mechanical energy conversion efficiency in our conductive liquid‐solid composited PVDF‐TrFE film. The high energy conversion efficiency is further illustrated in Figure 3g, Videos S1 and S2 (Supporting Information). 63 commercial blue LED bulbs in series were lit up by 0.06 g GaIn droplet filled device with palm tapping. Moreover, the stability of the as‐prepared PEH was tested by 36 000 continuous impacting/releasing cycles at a frequency of 30 Hz with 10 N tapping force and the result is shown in Figure 3h. The output can remain stable at 120±10 V without obvious deterioration manifesting the excellent mechanical stability of the PEHs. Meanwhile, the cross‐sectional SEM image of GaIn NDs/PVDF‐TrFE film before and after 36 000 impacting/releasing cycles is shown in Figure S9 (Supporting Information). The wrinkles can be observed in the lower part of the film after 36 000 cycles due to the feature of PVDF‐TrFE polymer. However, the dense and compact characteristics without cracks at the interface between GaIn droplet fillers and PVDF‐TrFE matrix in Figure S9b (Supporting Information) verified the satisfactory mechanical stability and reliability of the GaIn NDs/PVDF‐TrFE PEH, which is due to the well‐matched continuous liquid‐solid interface of GaIn and PVDF‐TrFE. The excellent durability of the device was supported by the maintenance of 200 V output voltage 60 days later by 12 N tapping force with a frequency of 30 Hz (as illustrated in Figure 3i).

**Figure 4:**
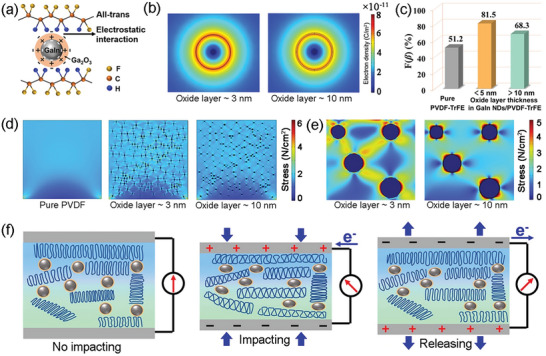
a) Schematic illustration of GaIn ND filler enabled the formation of *β* phase of PVDF‐TrFE. b) COMSOL simulations of the electron density of GaIn@Ga_2_O_3_ nanodroplet with the oxidation layer thickness of 3 and 10 nm. c) Variation of polar *β* phase content by the oxidation layer thickness. d) COMSOL simulations of the stress distribution in pure PVDF, GaIn NDs/PVDF‐TrFE with oxide layer thickness of 3 and 10 nm. e) Enlarged view of stress distribution with the thickness of 3 and 10 nm, respectively. f) Schematics presenting the piezoelectric output of GaIn NDs/PVDF‐TrFE based PEH during impacting/releasing process.

In our work, the prominent mechanical energy harvesting behavior of the GaIn NDs/PVDF‐TrFE based PEHs originates from the increased content of *β* phase and the enhanced piezoelectric property of the composite film by the filling of GaIn liquid nanodroplet. **Figure** **4**a schematically described the mechanism of the formation process of the *β* phase in LM/PVDF‐TrFE composite. GaIn NDs were covered by Ga_2_O_3_ oxide layer in the ultrasonic process that can be regarded as a dielectric layer. Since the surface of the liquid metal droplet has electron‐rich characteristics, the positive charges were induced on the inner surface of Ga_2_O_3_ layer toward GaIn droplet while equal positive charges accumulated on the surface toward PVDF‐TrFE matrix. Due to the electrostatic interaction, the positive ‐CH_2_ groups of PVDF‐TrFE tended to turn to GaIn droplets resulting in the alignment of PVDF‐TrFE chains and promoting *β* phase content.^[^ ^18^ ^]^ Except for the role of boosting *β* phase content of PVDF‐TrFE composite film, the presence of GaIn droplets as a conductive filler are also helpful for amplifying the local electric field benefitting the higher polarizability of PVDF‐TrFE and corresponding piezoelectricity.^[^ ^19^ ^]^

As discussed above, the oxidation layer thickness has shown a significant effect on the *β* phase content of PVDF‐TrFE composite film. It is generally accepted that Ga_2_O_3_ shell layer was naturally oxidized from GaIn core as Ga is easier to be oxidized, thus then the electron density gradient would be generated by the change of its thickness.^[^ ^20^ ^]^ To evaluate the effect of the oxide layer thickness theoretically, finite element simulations of the surface electron density were established. The diameter of the liquid metal sphere is 100 nm, and the thickness of the oxidation shell layer is set as 3 and 10 nm, respectively (the details of the simulation are described in the Supporting Information). As shown in Figure 4b, it turned out that the higher electron density was induced near the surface of the oxidation layer with a thickness of 3 nm. In order to verify the simulation results, Zeta potential measurements for GaIn@Ga_2_O_3_ NDs with the oxidation layer thickness of 3 and 10 nm were performed using the electrophoretic light scattering method (Malvern Zetasizer Nano ZS90) to further explore the surface charge density of the nanodroplets with the different oxidation layer thickness. Zeta potential of 18.2 mV was observed for the GaIn@Ga_2_O_3_ NDs with the oxidation layer thickness of 10 nm. The GaIn@Ga_2_O_3_ NDs with the oxidation layer thickness of 3 nm carried a higher Zeta potential of 24.0 mV, which demonstrated its high surface charge density and better dispersion (Figure S10, Supporting Information).^[^ ^21^ ^]^ Additionally, the change in surface potentials of GaIn@Ga_2_O_3_ NDs with the oxidation layer thickness of 3 and 10 nm was measured using Kelvin probe force microscopy (KPFM) as shown in Figure S11 (Supporting Information). It can be found that the surface potential of GaIn@Ga_2_O_3_ NDs with the oxidation layer thickness of 10 nm is in the range of −330–370 mV. The value increased to ≈‐430 mV for the nanodroplets with the oxidation layer thickness of 3 nm indicating the work function decreases for the thinner oxidation layer. The lower surface potential indicates a higher density of negative charges on the surface of GaIn@Ga_2_O_3_ nanodroplets.^[^ ^20^, ^22^ ^]^ Therefore, more positive‐charged ‐CH_2_ groups of PVDF‐TrFE tend to be the all‐trans alignment, and higher *β* phase content could be then obtained (Figure 4c).^[^ ^23^ ^]^ Similar phenomena were also reported in the core‐shell structure such as Fe@Fe_3_O_4_ which manifested the great influence on the electron density of the shell surface.^[^ ^24^ ^]^ However, the GaIn NDs/PVDF‐TrFE composite film would break down in the subsequent high voltage polarization process if the Ga_2_O_3_ shell is too thin, while a thicker Ga_2_O_3_ shell would bring out lower surface electron density.^[^ ^20^, ^25^ ^]^

Furthermore, it should be noteworthy that the electrical output performance of the PEH device not only affected by piezoelectric coefficient but also by the stress of the film.^[^ ^26^ ^]^ In order to theoretically analyze the influence of the LM droplets on the force transfer efficiency, the stress distributions in the pure PVDF film and liquid droplets composited PVDF film with Ga_2_O_3_ layer thickness of 3 and 10 nm are simulated (the details are described in the Supporting Information). And it can be found from Figure 4d that the liquid droplets composited PVDF film experiences apparently larger stress than the pure PVDF film under the same compressive load, which is in favor of effectively upgrading the output voltage of the GaIn NDs/PVDF‐TrFE PEH. In addition, a more obvious stress concentration around the LM particle with Ga_2_O_3_ layer thickness of 10 nm can be observed by comparing Figure 4e, which would result in an unstable interface between LM nanofiller and PVDF‐TrFE matrix. Actually, the fracture failure of the Ga_2_O_3_ shell and the wrecked interface was observed in the area marked with a white dashed line in oxygen element distribution(in Figure S5, Supporting Information). These results demonstrate that another merit of the proposed GaIn NDs/PVDF‐TrFE composites is regulation of the liquid‐solid/conductive‐dielectric interface could further optimize the electromechanical conversion performance of piezoelectric films.

Therefore, there is a critical thickness of the shell to balance the properties between high polar active content and the stability of the composite film. In this work, the thickness of Ga_2_O_3_ shell was regulated by controlling the ultrasonic medium. When the Ga_2_O_3_ shell thickness was less than 5 nm, the high surface electron density helps the higher *β* phase content of ≈81.5%. In the case of the shell layer thicker than 10 nm, the *β* phase content is reduced to ≈68.3% owing to the relatively low surface electron density, as presented in Figure 4c. Additionally, the impressive high *d_33_* value and output voltage in this work also benefit from the local electric field stemming from GaIn conductive filler that further improves the polarizability and corresponding piezoelectric property of GaIn NDs/PVDF‐TrFE composite membrane.^[^ ^27^ ^]^

Accordingly, the working mechanism of GaIn NDs/PVDF‐TrFE PEH for harvesting mechanical energy to generate electric output can be schematically shown in Figure 4f. There is no piezoelectric output in the case of the absence of external compacting force. When a compacting force is applied to the device, GaIn droplets deform with external force and play the role of transferring the stress in the composited film. PVDF‐TrFE undergoes a compressive deformation and the polarization of PVDF‐TrFE molecules resulting in the accumulation of free charges on the top surface of the composite film and thereby inducing the potential difference between the two electrodes. Therefore, electrons flow between the upper and lower electrodes and the piezoelectric output current is obtained. In the case of releasing force, the deformation of GaIn droplets and PVDF‐TrFE recovers. As a result, the polarization is weakening and the decline of the free charge's number on the top surface leads to a reversed piezoelectric output current. The adaptive deformability of liquid fillers offers a continuous and flexible interface in such liquid‐solid construction during each compacting‐releasing process to guarantee the long‐term stability of both the internal structure and output performance.

**Figure 5:**
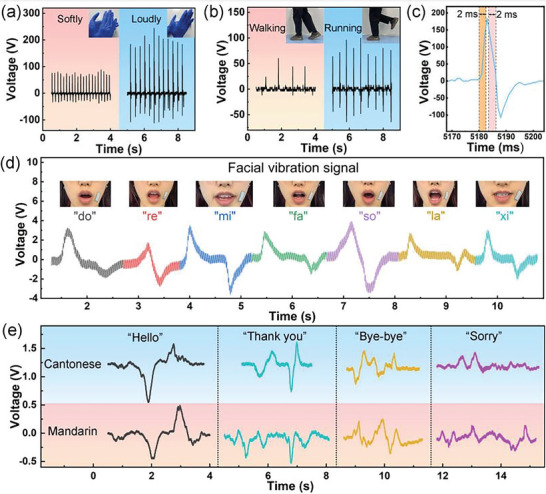
Practical applications of the liquid GaIn droplets filler based PEH to harness the energy from limbs of the human body or self‐powered sensor. Output voltage signals of the device mounted a) in the palm with clapping softly and loudly, b) on the heel of the shoe‐pad with walking and running, c) Enlarged views of electrical signal for estimating the response and recovery time for detecting limbs activity of human. Sensing the performance of the GaIn NDs/PVDF‐TrFE based device by attaching it to the corner of the adult volunteer's mouth. Dynamic voltage profiles of vibration signal during speaking d) seven notes of “do”, “re”, “me”, “fa”, “so”, “la”, “xi”, e) “Hello”, “Thank you”, “Bye‐bye”, “Sorry” in Mandarin and Cantonese.

To explore the possibility of the liquid conductive droplet fillers‐based devices as the ideal candidate for non‐uniform external force‐based biomechanical energy and self‐powered sensor, the optimal PEH device was applied to harness the energy from the large amplitude motions of limbs and detect the minor muscle movements from frowning or speaking. **Figure** **5**a illustrates the resultant output voltage generated by hand activity, which achieved ≈80 V for clapping gently and ≈214 V for clapping loudly. When the device was attached to the heel of the shoe pad to harness the output voltage signals from walking and running, the yielded maximum voltage is ≈60 and ≈96 V, as shown in Figure 5b. In addition, Figure S12a (Supporting Information) also represents the output voltage signals by finger bending softly and sharply, with signal peak values of ≈15 and ≈35 V, respectively, demonstrating that the GaIn NDs/PVDF‐TrFE based PEH can stably harvest the energy from the limb's movement. Moreover, the enlarged views of the electrical signal are depicted in Figure 5c and Figure S12b (Supporting Information) to estimate the response/recovery time of the tactile sensor based on GaIn NDs/PVDF‐TrFE composited film. Response and recovery times are defined as the time period of the output voltage reaching 90% of the maximum value and attenuating by 90% from the maximum level, respectively. It can be found that the response/recovery time reaches the millisecond level illustrating the fast response of PVDF‐based piezoelectric sensor. A short response/recovery time of 2 ms/2 ms was achieved during clapping loudly mode, which was significantly faster than the reported time of 33 ms/20 ms by punching a CNT/CsPbBr_3_/PVDF‐TrFE nanofibers device and the response time of 80 ms for the solid filler based piezoelectric device.^[^ ^28^ ^]^ The sharp response characteristics illustrate the fast stress transfer ability in GaIn droplet‐filled PVDF‐TrFE film.

The feasibility of GaIn droplet fillers‐based devices for sensing facial expression or tracking facial muscle movement was also explored by attaching it to the face. By comparing the real‐time output voltage profiles in response to facial muscle movement in Figure 5d,e and Figure S12c–e (Supporting Information), the output voltage is only a few volts, which is obviously lower than that signal from limb movement. However, the dynamic voltage profiles originated from speaking seven musical notes from “do” to “xi” in Figure 5d presented obvious distinguished wave patterns corresponding to each note that can be supposed as effective evidence for voice recognition. Therefore, the response wave profiles from facial muscle movements provided compelling evidence that the high sensitivity for low stress of GaIn droplet filled PVDF‐TrFE film‐based sensor. More interestingly, when our volunteer is speaking words in Mandarin and Cantonese, the reflected real‐time signals from the facial muscle movement near the mouth corner exhibited wide discrepancies not only in shape but also in frequency since the two dialects have different tones and rhythms (Figure 5e). These results also signified that our device might be designed as a facial muscle training instrument for guiding autistic patients to practice phonation.

## 3 Conclusion

A novel flexible piezoelectric composited film consisting of liquid GaIn nanodroplet‐PVDF‐TrFE polymer interface was actualized in this paper. In particular, the liquid GaIn nanofillers not only behave as conductive elements to strikingly improve the polarization of PVDF‐TrFE by the local electric field, but also act as a perfect bridge between fillers and PVDF‐TrFE matrix to significantly promote the *β* phase content, which is attributed from the electrostatic interaction between positive ‐CH_2_ groups of PVDF‐TrFE and the induced negative charge on the surface of Ga_2_O_3_ shell of the GaIn droplet. The thickness of the Ga_2_O_3_ shell played a critical role in the surface electron density and polar *β* phase content by controlling the ultrasonic solution medium. It is manifested that the much higher piezoelectric coefficient (*d_33_* ≈51.1 pC N^−1^) and remarkable maximum output voltage ≈212 V were achieved with an optimized mass of GaIn LM nanofillers, which outperformed most of the previous experimental results of piezoelectric nanogenerator. The output kept stable without obvious deterioration after 36 000 cycles and 60 days verified the satisfactory mechanical durability and reliability of the GaIn NDs/PVDF‐TrFE PEH. Attractively, this conductive filler PEH was demonstrated to be competent to harness the human limb motion but also to monitor the real‐time facial muscle signal during pronunciation for potential application of guiding autistic patients to practice phonation. This work verified that our rationally designed organic molecule engineered conductive liquid filler based piezoelectric polymer film opens a facile and effective approach for the next autonomous rehabilitation medicine training system.

## 4 Experimental Section

### Synthesis of GaIn NDs/PVDF‐TrFE Composited Films

GaIn NDs/PVDF‐TrFE composited films were prepared by solution blending with eutectic GaIn alloy droplet of DMF solution and PVDF‐TrFE DMF solution. First, 0.5 g PVDF‐TrFE pellets (PVDF/PTrFE = 70/30 mol%, *Mw*≈450,000 g mol^−1^, Piezotech Arkema Co) was dissolved in 7 mL DMF solvent by magnetic stirring for 2 h at room temperature. The GaIn NDs were obtained by an ultrasonic homogenizer (JY92‐IIN). Briefly, liquid GaIn alloy with different masses (0.02, 0.04, 0.06, 0.08, and 0.10 g) was added to 7 mL DMF solvent in a 20 mL glass vial that was placed in an ice bath. A probe (5 mm diameter) was immersed into the solution, which was sonicated at 60% power using a burst mode (on/off, 1/2 s) for 30 min. The temperature of the ice bath was maintained at ≈0–3 °C during the sonication process. Then, the DMF solution with GaIn droplets was obtained. To prepare the GaIn NDs/PVDF‐TrFE composite film, the GaIn droplet DMF solution and DMF solution of PVDF‐TrFE were mixed and sonicated for 30 min followed by keeping stirring for 24 h to form a suspension. After that, the suspension was poured into a self‐made quartz vessel and kept at 80 °C overnight. The final film was annealed at 145 °C for further crystallization, and GaIn NDs/PVDF‐TrFE layer was peeled off from the bottom of the quartz vessel.

### Fabrication of the GaIn NDs/PVDF‐TrFE based PEHs

The GaIn NDs/PVDF‐TrFE composited film was cut into 2 × 3 cm^2^ square pieces. Then, two pieces of aluminium tape were attached to the two sides of the composite film as electrodes to form a sandwich stack configuration. Subsequently, the device was kept in an oil bath and poled by applying a direct electric field of 30 kV mm^−1^ at 130 °C for 6 h. Finally, two pieces of PI films were attached on each side of the device and compressed to complete the fabrication process of GaIn NDs/PVDF‐TrFE PEH as shown in Figure S1a (Supporting Information). This kind of packaging contributes to hindering the influence of triboelectricity between different films on the piezoelectricity and protecting the device from a harmful ambient environment. The digital photograph in Figure S1b (Supporting Information) shows the good flexibility of the device. To synthesize the control W‐GaIn NDs/PVDF‐TrFE based PEH, liquid eutectic GaIn alloy was fabricated by ultrasonic dispersion in deionized water by a similar procedure and the corresponding droplets are denoted as W‐GaIn NDs.

### Characteristics

The morphologies of the GaIn LM particles and GaIn NDs/PVDF‐TrFE films were characterized by a field emission scanning electron microscope (FESEM, JEOL JSM7100F) and transmission electron microscope (TEM, JEOL2100). X‐ray diffraction (XRD) patterns were recorded by a Bruker D8 Advance diffractometer with Cu‐K*α* radiation. Fourier‐transform infrared spectroscopy (FT‐IR, VERTEX 70 V) was used to characterize the phase composition of GaIn NDs/PVDF‐TrFE composited films in the 700–1700 cm^−1^ using attenuated total reflectance (ATR) mode. Further, the element type of powder samples was detected by X‐ray photoelectron spectroscopy (XPS, ThermoFisher Scientific Escalab 250Xi). The dielectric constant was measured by LCR meter (TH2827) with a frequency range of 100 Hz–1 MHz and Precision Multiferroic Materials Analyzer (Radiant Inc. US) was utilized to measure the polarization‐electrical field (*P*‐*E*) hysteresis loops at 100 Hz. Zeta potential and surface potential profile measurements for GaIn@Ga_2_O_3_ NDs were performed using the electrophoretic light scattering method (Malvern Zetasizer Nano ZS90) and Kelvin probe force microscopy (KPFM, Bruker Dimension Icon), respectively. The energy harvesting of PEH was tested by a vibration exciter as the mechanical stimulation source was controlled by a function signal generator with a power amplifier. The output voltage and current signals of the PEHs were recorded by a digital oscilloscope (MDO 3024), charge amplifier (SA 1804B), and electrometer (Keithley 2611).

## Conflict of Interest

The authors declare no conflict of interest.

## Supporting information

Supporting Information

Supplemental Video 1

Supplemental Video 2

## Data Availability

The data that support the findings of this study are available in the supplementary material of this article.
