# Supplementary material for: Giant Piezoelectric Output and Stability Enhancement in Piezopolymer Composites with Liquid Metal Nanofillers
Source: Adv Sci (Weinh). 2023 Sep 13;10(36):2304096. doi: 10.1002/advs.202304096 (PMC10754131; doi:10.1002/advs.202304096)
Supplement: Supplementary file 1 — Supporting Information [file ADVS-10-2304096-s002.pdf]

ADVANCED SCIENCE
Open Access

Supporting Information

for *Adv. Sci.*, DOI 10.1002/advs.202304096

Giant Piezoelectric Output and Stability Enhancement in Piezopolymer Composites with Liquid Metal Nanofillers

Jingyan Liu, Shi Zeng, Mingrui Zhang, Juan Xiong, Haoshuang Gu, Zhao Wang, Yongming Hu, Xianghui Zhang, Yi Du and Long Ren**

Supporting Information

Giant piezoelectric output and stability enhancement in piezopolymer composites with liquid metal nanofillers

Jingyan Liu, Shi Zeng, Mingrui Zhang, Juan Xiong*, Haoshuang Gu, Zhao Wang, Yongming Hu, Xianghui Zhang, Yi Du and Long Ren*

Supplementary Experimental Details:

Finite element simulation of the electron density

To calculate the electron density on the surface of the oxidation layer, the geometric model of the electrostatic field was constructed. The assignment of electron density on the surface of the LM droplet was 1×10^{-3} C/m^2, notes as Q. Therefore, the electron density, σ, on the surface can be calculated according to the formula:

$$\sigma = \frac{Q}{4\pi r^2}$$

Here, r is the diameter of the LM nanodroplet including GaIn core and Ga_2O_3 shell layer. The diameter of GaIn core was 100 nm and the thickness of Ga_2O_3 shell layer was 3 nm and 10 nm, which is consistent with the TEM results.

Finite element simulation of the stress distribution

To simulate the stress distribution of PVDF matrix, the layer thickness is 60 μm corresponding to the SEM results. The composited film consists of randomly dispersed liquid droplets with a diameter in the range of 100~500 nm. A compressive force of 30 MPa is applied on the upper surface of the film, whereas the lower surface was fixed.

Supplementary Figures:

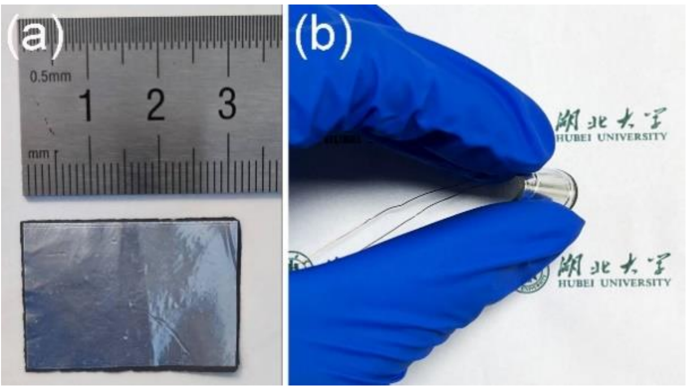

Figure S1. Photographs of (a) GaIn NDs/PVDF-TrFE film piece, (b) corresponding PEH device.

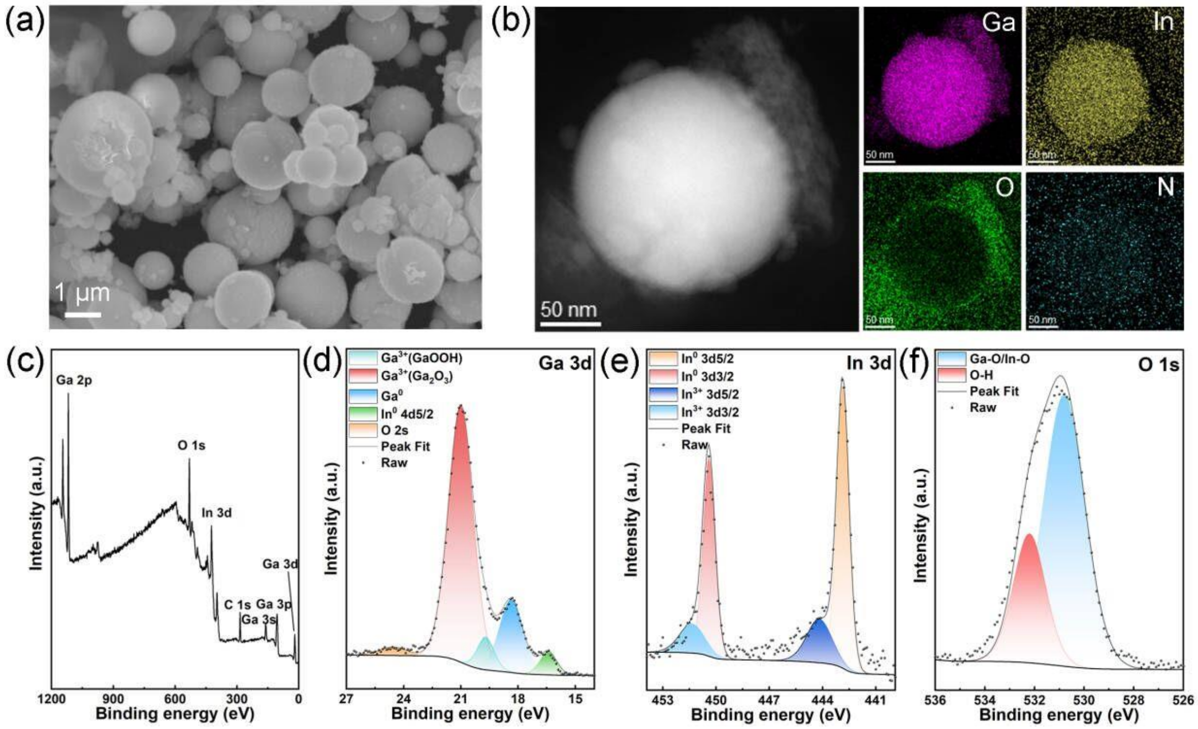

Figure S2. (a) SEM image, (b) HAADF-STEM image and EDS mapping images, XPS spectra of W-GaIn NDs: (c) survey, (d) Ga 3d, (e) In 3d and (f) O 1s.

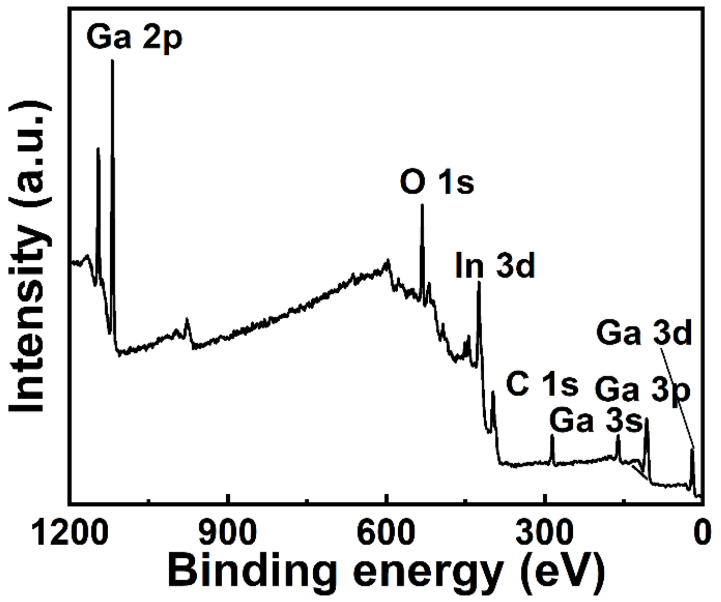

Figure S3. The survey XPS spectra of GaIn NDs

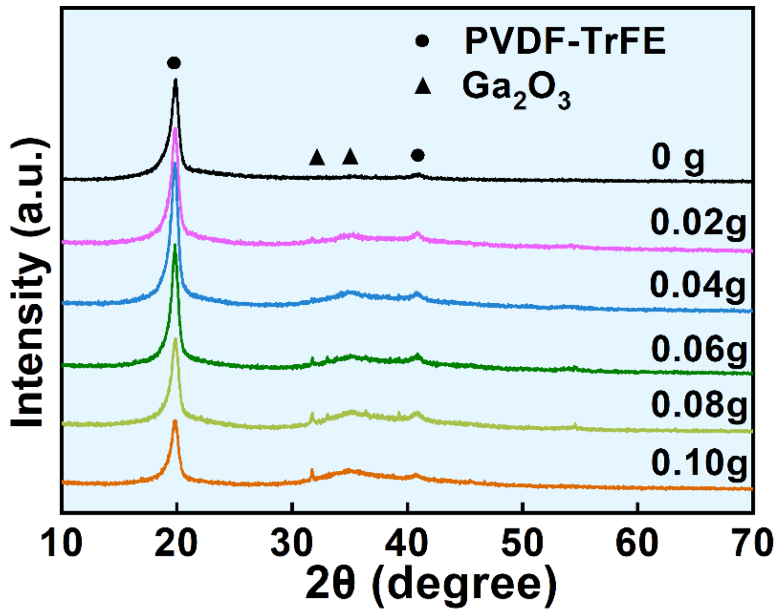

Figure S4. XRD patterns of GaIn NDs/PVDF-TrFE composite films with different mass of GaIn NDs.

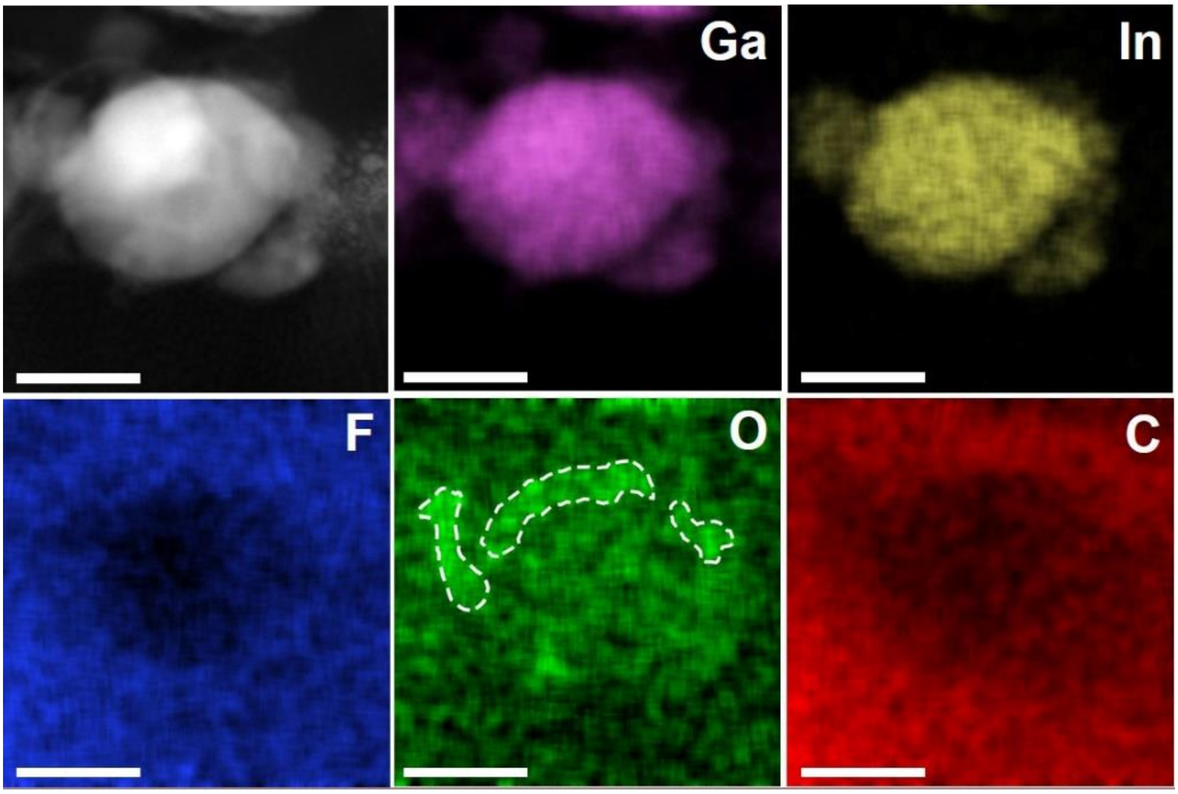

Figure S5. HAADF-STEM image of W-GaIn NDs/PVDF-TrFE film: representative image of W-GaIn ND in PVDF-TrFE matrix and EDS mapping of corresponding elemental distributions. Scale bars, 100 nm.

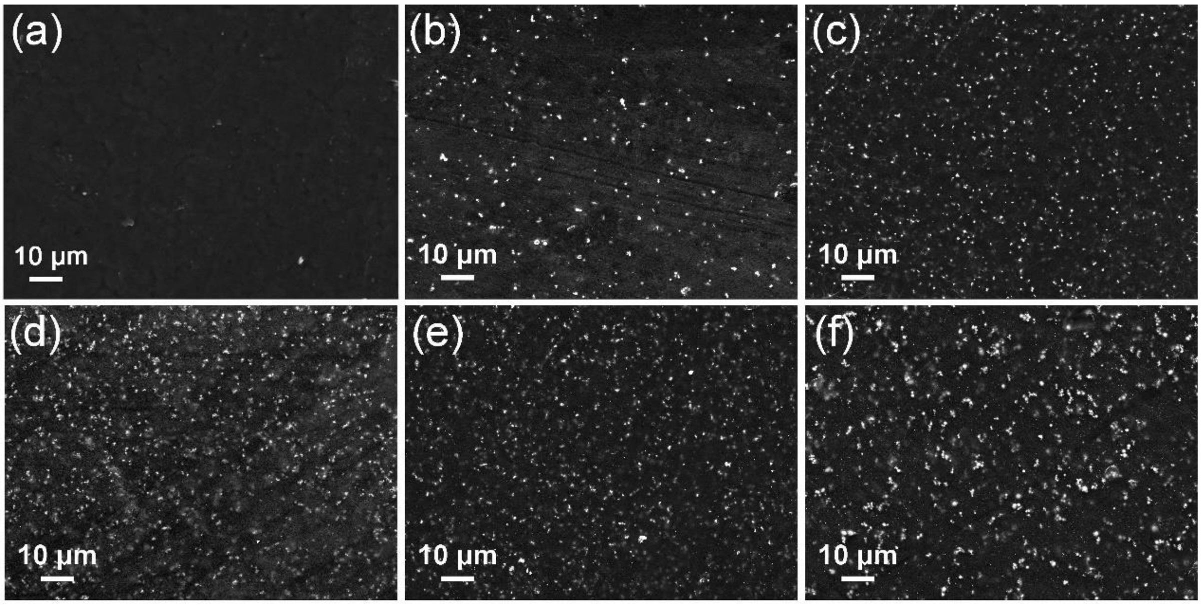

Figure S6. Surface SEM images of GaIn NDs/PVDF-TrFE with different weights of GaIn. (a) without GaIn NDs, (b) 0.02 g, (c) 0.04 g, (d) 0.06 g, (e) 0.08 g, (f) 0.10 g.

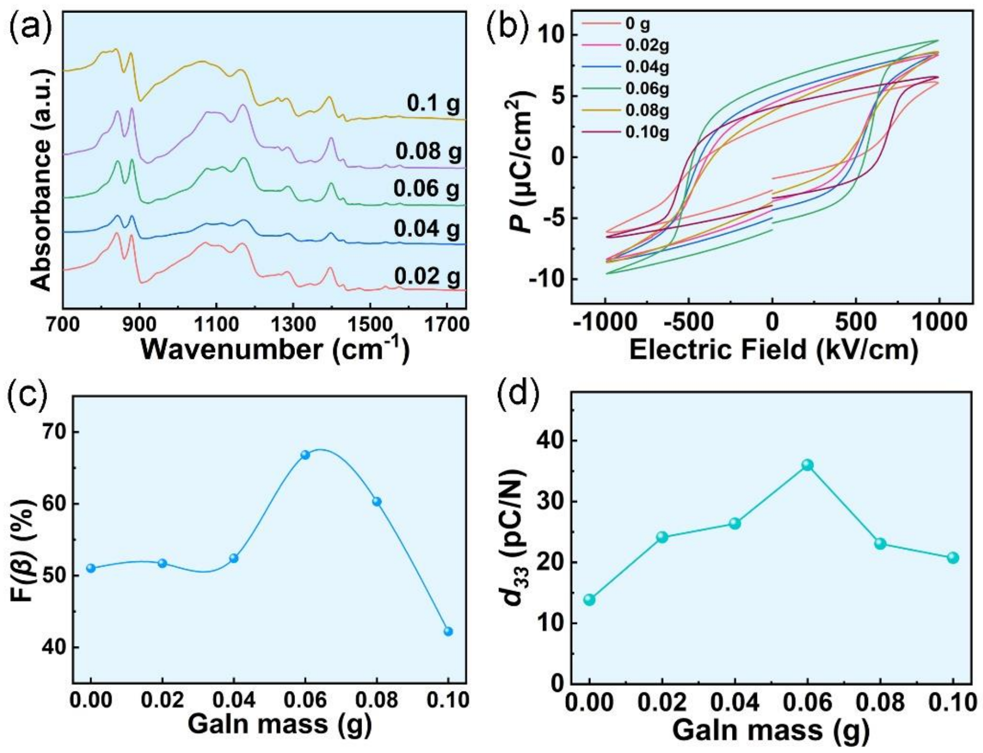

Figure S7. (a) FT-IR spectra, (b) *P-E* loops, (c) the variation of calculated β phase content and (d) d_{33} of W-GaIn NDs/PVDF-TrFE films with different mass of W-GaIn NDs.

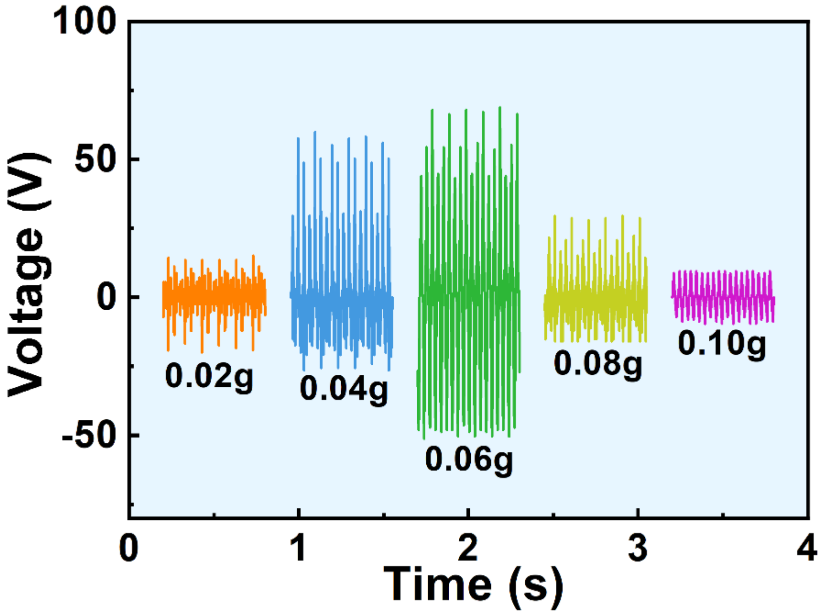

Figure S8. The output voltage of the W-GaIn NDs/PVDF-TrFE PEHs based on different mass of W-GaIn NDs under 12 N tapping force.

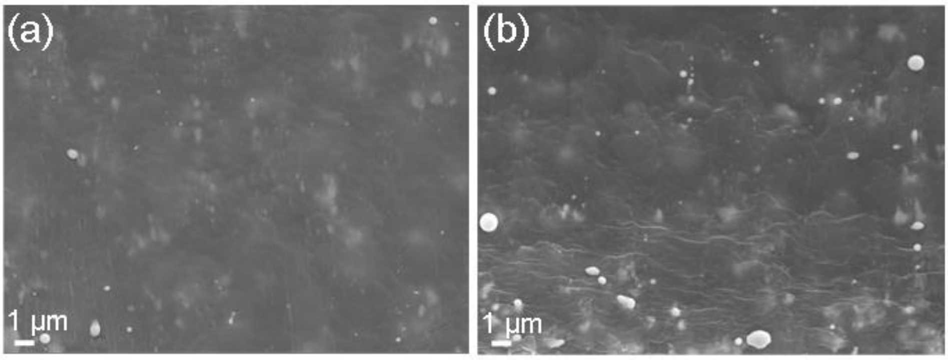

Figure S9. The cross-sectional SEM images of GaIn NDs/PVDF-TrFE PEH (a) before and (b) after 36,000 times tapping under 10 N tapping force with 30 Hz.

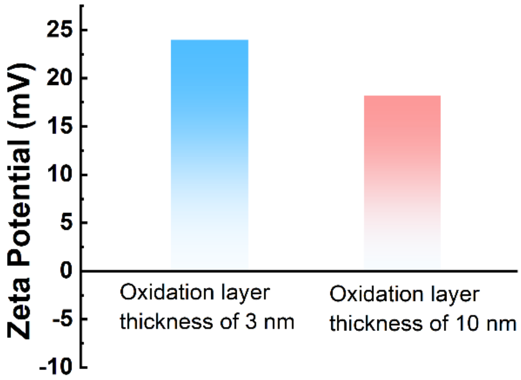

Figure S10. Zeta potential of GaIn@Ga_2O_3 NDs with the oxidation layer thickness of 3 nm and 10 nm.

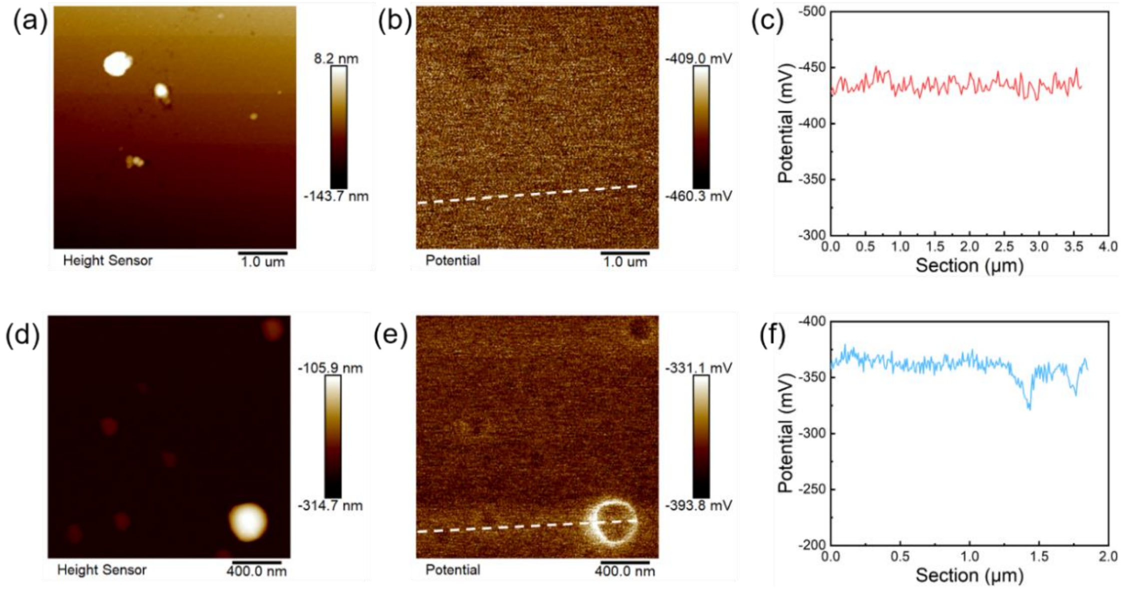

Figure S11. AFM image, KPFM image and corresponding line scanning surface potential profile of GaIn@Ga_2O_3 NDs with the oxidation layer thickness of 3 nm (a, b, c) and 10 nm (d, e, f).

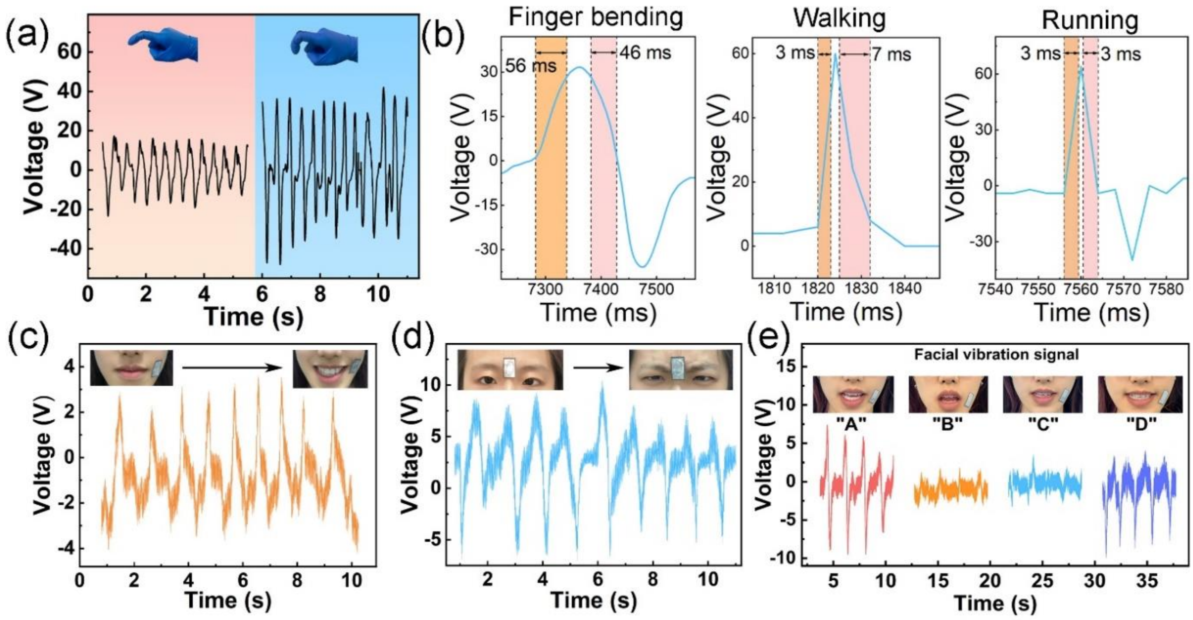

Figure S12. Output voltage signals of the device mounted (a) on finger with slight and sharp bending. (b) Enlarged views of electrical signal for estimating the response and recovery time for detecting limbs activity of human. Real-time output voltage profile in response to (c) smiling and (d) frowning. (e) Dynamic voltage profiles of vibration signal of mouth corner during speaking letters of “A”, “B”, “C”, “D”.

Supplementary video

Video S1. 60 LEDs glowing by the GaIn NDs/PVDF-TrFE PEH under repeated human palm beating.

Video S2. 63 blue LEDs glowing by the GaIn NDs/PVDF-TrFE PEH under repeated human palm beating.
